# Supplementary material for: Safety, Tolerability, and Immunogenicity of V160, a Conditionally Replication-Defective Cytomegalovirus Vaccine, in Healthy Japanese Men in a Randomized, Controlled Phase 1 Study
Source: Antibodies (Basel). 2023 Mar 10;12(1):22. doi: 10.3390/antib12010022 (PMC10045923; doi:10.3390/antib12010022)
Supplement: Supplementary file 1 [file antibodies-12-00022-s001.zip › antibodies-2237111-supplementary-final.docx]

**
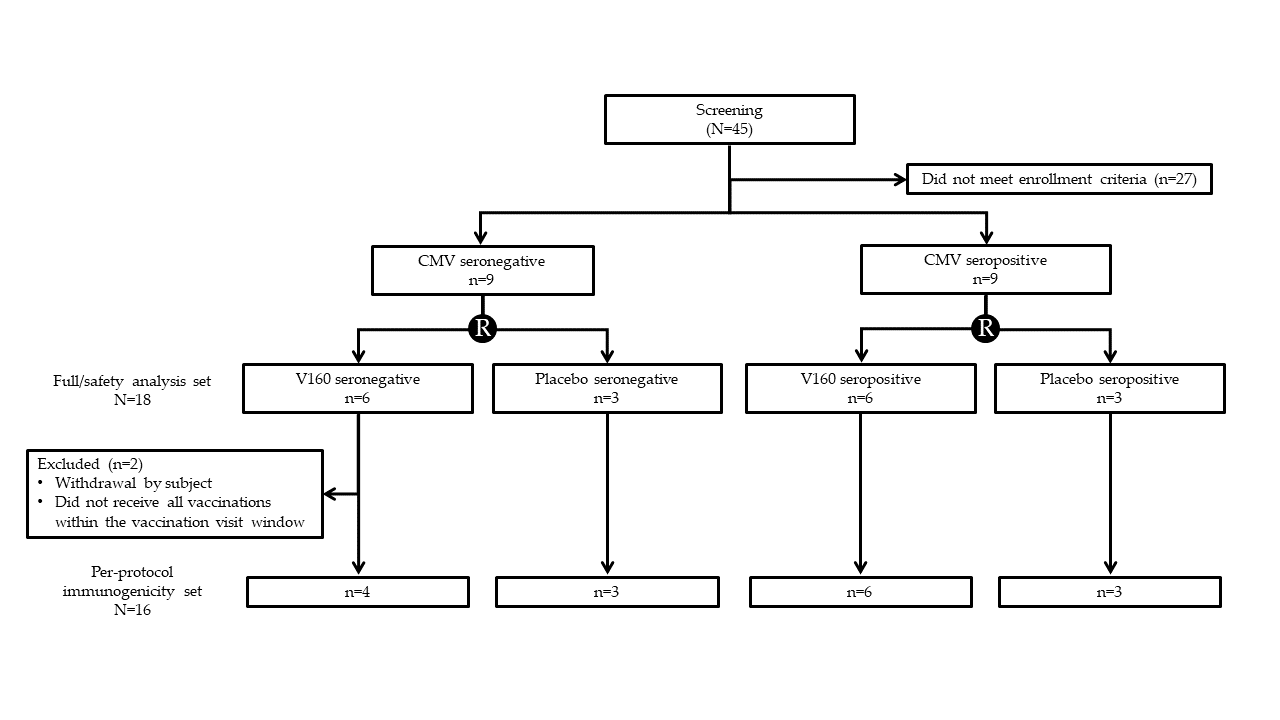
**

**Figure S1.** CONSORT flow diagram. CONSORT: Consolidated Standards of Reporting Trials; R, randomization.

**Table S1.** Participants with any positive viral detection in plasma by CMV serostatus and study visit after dose 1 (A) and by virus type (B)

| **A** | | | | | | | | | | | | | | |
| --- | --- | --- | --- | --- | --- | --- | --- | --- | --- | --- | --- | --- | --- | --- |
| Timepoint | V160 seropositive | | | Placebo seropositive | | | | V160 seronegative | | | | Placebo seronegative | | |
|  | n/T | | n (%) | n/T | | n (%) | | n/T | n (%) | | | n/T | | n (%) |
| Day 1 (predose) | 6 (6) | | 0 (0) | 3 (3) | | 0 (0) | | 6 (6) | 0 (0) | | | 3 (3) | | 0 (0) |
| Day 1, 0 min | 6 (6) | | 0 (0) | 3 (3) | | 0 (0) | | 6 (6) | 1 (16.7) | | | 3 (3) | | 0 (0) |
| Day 1, 3 h | 6 (6) | | 0 (0) | 3 (3) | | 0 (0) | | 6 (6) | 0 (0) | | | 3 (3) | | 0 (0) |
| Day 3 | 6 (6) | | 6 (100) | 3 (3) | | 0 (0) | | 6 (6) | 6 (100) | | | 3 (3) | | 0 (0) |
| Day 7 | 6 (6) | | 0 (0) | 3 (3) | | 0 (0) | | 6 (6) | 0 (0) | | | 3 (3) | | 0 (0) |
| Day 14 | 6 (6) | | 0 (0) | 3 (3) | | 0 (0) | | 6 (6) | 0 (0) | | | 3 (3) | | 0 (0) |
| **B** | | | | | | | | | | | | | | |
| Timepoint | V160 seropositive (N = 6) | | | Placebo seropositive (N = 3) | | | V160 seronegative (N = 6) | | | | Placebo seronegative (N = 3) | | | |
|  | V160 type, % (m/n) | Wild type, % (m/n) | | V160 type, % (m/n) | Wild type, % (m/n) | | V160 type, % (m/n) | | | Wild type, % (m/n) | V160 type, % (m/n) | | Wild type, % (m/n) | |
| Day 1 (predose) | 0 (0/6) | 0 (0/6) | | 0 (0/3) | 0 (0/3) | | 0 (0/6) | | | 0 (0/6) | 0 (0/3) | | 0 (0/3) | |
| Day 1, 0 min | 0 (0/6) | 0 (0/6) | | 0 (0/3) | 0 (0/3) | | 16.7 (1/6) | | | 0 (0/6) | 0 (0/3) | | 0 (0/3) | |
| Day 1, 3 h | 0 (0/6) | 0 (0/6) | | 0 (0/3) | 0 (0/3) | | 0 (0/6) | | | 0 (0/6) | 0 (0/3) | | 0 (0/3) | |
| Day 3 | 50.0 (3/6)^a^ | 16.7 (1/6)^a^ | | 0 (0/3) | 0 (0/3) | | 100 (6/6) | | | 0 (0/6) | 0 (0/3) | | 0 (0/3) | |
| Day 7 | 0 (0/6) | 0 (0/6) | | 0 (0/3) | 0 (0/3) | | 0 (0/6) | | | 0 (0/6) | 0 (0/3) | | 0 (0/3) | |
| Day 14 | 0 (0/6) | 0 (0/6) | | 0 (0/3) | 0 (0/3) | | 0 (0/6) | | | 0 (0/6) | 0 (0/3) | | 0 (0/3) | |

^a^ Since the viral load in the other 2 participants of seropositive participants were low, these samples were not discriminated vaccine-type or wild type.CMV, cytomegalovirus; m = Number of subjects with at least one positive sample for viral detection in plasma; n = Number of subjects with at least one sample collected; T, total number of participants.
